# Effects of Active Video Games in Patients With Cancer: Systematic Review

**DOI:** 10.2196/45037

**Published:** 2023-05-26

**Authors:** Romane Peyrachon, Amélie Rébillard

**Affiliations:** 1 Movement, Sport and Heath Sciences laboratory M2S-EA7470 University of Rennes 2 Rennes France; 2 Institut Universitaire de France Paris France

**Keywords:** exergaming, cancer, physical activity, fatigue, endurance, strength, adjuvant therapy, cancer therapy, cancer treatment, video games, digital health intervention, cancer patient

## Abstract

**Background:**

Physical activity (PA) is now considered an adjuvant therapy in cancer treatment; nevertheless, multiple barriers could reduce PA engagement during treatment. Active video games (AVGs) lead to the achievement of mild- to moderate-intensity PA and represent a promising tool for regular movement and exercise.

**Objective:**

This paper aims to review the current literature and provide updated content on the physiological and psychological effects of AVG-based interventions in patients with cancer undergoing treatment.

**Methods:**

Four electronic databases were investigated. Studies reporting on AVG interventions delivered to patients undergoing treatment were included. A total of 21 articles (17 interventions) were identified for data extraction and quality assessment.

**Results:**

A total of 362 patients with cancer participated in the studies (number of participants 3-70). The majority underwent treatment for breast, lung, prostate, hematologic, or oral or laryngeal cancer. The types and stages of cancer varied in all studies. Participants ranged in age from 3 to 93 years. Four studies included patients with pediatric cancer. The duration of interventions ranged from 2 to 16 weeks, with a minimum of 2 sessions per week and a maximum of 1 daily session. Sessions were supervised in 10 studies, and 7 included home-based interventions. AVG interventions improved endurance, quality of life, cancer-related fatigue, and self-efficacy. Effects were mixed on strength, physical function, and depression. AVGs did not affect activity level, body composition, or anxiety. Compared with standard physiotherapy, physiological effects were lower or similar, and psychological effects were higher or similar.

**Conclusions:**

Overall, our results suggest that AVGs can be recommended to patients undergoing cancer treatment, given the physiological and psychological benefits. When AVGs are proposed, supervision of the sessions should be considered as it can limit dropouts. In the future, it is important to develop AVGs that combine endurance and muscle strengthening, with the possibility of achieving moderate to high exercise intensity, depending on the physical abilities of the patients, as indicated in the World Health Organization’s recommendations.

## Introduction

Physical activity (PA) is now considered an adjuvant therapy in cancer treatment [1]. This promising strategy provides psychological (decreased cancer-related fatigue [CRF], decreased anxiety or depression, and improved quality of life [QoL] [2-6]) and physiological benefits (improved fitness, improved muscle strength and function, and normalization of body composition [4,7,8]) in patients with cancer and cancer survivors. Interestingly, a growing body of evidence now suggests that PA is associated with a reduction in cancer-specific mortality [9-11]. Therefore, experts recommended that patients with cancer be as physically active as possible and limit sedentary time [12]. An effective exercise prescription should include moderate-intensity aerobic exercise training at least 3 times per week for 30 minutes combined with 2 sessions of resistance training per week [4].

Unfortunately, 93% of patients with cancer are insufficiently active [13]. Multiple barriers have been identified to support this finding. They can be organizational (schedule of care, location of practice, and availability of therapists and venues) [14,15], physical (pain, lymphedema, CRF, or treatment side effects [14,16-18]), or psychological and social. Abo et al [14] show that the main individual limitations of patients with cancer are lack of motivation and emotional burden. Feeling unable to perform physical exercise or fear of injury is also reported [16]. Therefore, solutions are needed to reconnect patients with cancer to PA and keep them engaged.

New technologies have emerged as a promising tool for regular movement and exercise. Active video games (AVGs), also known as exergames (eg, Just Dance, Wii Fit Plus, and Beat Saber), are becoming increasingly accessible [19]. They are defined as engaging, safe, and fun games in which the players interact in the environment through their movements [19-21]. A few studies have investigated the impact of AVGs in promoting PA in healthy populations [22] or those with disease [23], showing that AVGs lead to the achievement of mild- to moderate-intensity PA [23,24]. These preliminary results suggest that AVGs can help patients reach PA recommendations and thus could provide several health benefits [23,25-27]. Importantly, as described by Tough et al [28], adherence to the AVG intervention is greater than that to standard care in adults with a current or previous cancer diagnosis. Nevertheless, the lack of studies and heterogeneity of interventions and patients hinder conclusions about the real impact of AVGs on health.

In this context, the purpose of this paper is to review the current literature and provide updated content on the physiological and psychological effects of AVG-based interventions in patients with cancer undergoing treatment.

## Methods

### Study Design

This review was conducted in accordance with the PRISMA (Preferred Reporting Items for Systematic Review and Meta-Analysis) [29].

### Search Strategies

Four databases (MEDLINE, PubMed, SPORTDiscus, and Google Scholar) were investigated from inception to February 2023. Keywords were defined with the PICO method [30]. The search strategy was based on the following keywords and their associated synonyms: “Cancer,” “Active video game,” “Exergames,” “Virtual Reality,” “Physical activity,” and “Exercise.” There were no restrictions by date or study location. Additional articles were added manually by searching the references of included studies.

### Study Selection

Articles from different databases were combined into a single file, and duplicates were removed. Next, eligibility was assessed by a reviewer (RP) using a 2-step process. At any point, if there was any doubt, a second reviewer (AR) helped to decide.

First, the reviewer screened the title and abstracts of each article. Studies were considered for the second phase if the title or abstract indicated that the intervention was PA based on AVGs in human populations. No age restrictions were considered. The second phase consisted of a full-text review. The inclusion and exclusion criteria for the screening process are presented in Textbox 1.

**Inclusion and exclusion criteria for the study screening.**

**Inclusion criteria:**

- Article type

Clinical trials: research that compared the active video game intervention with healthy controls (ie, cohort and case studies), participants serving as their own control (ie, longitudinal evaluation), and usual physical activity (PA) program or care (ie, randomized control)

- Language

English and French

- Population

Patients with cancer undergoing treatments

- Intervention

Exergames, virtual reality to support PA, and chronic intervention (more than 1 week)

- Outcomes

Physiological or psychological outcomes were reported. Physiological outcomes included PA level, motor functions, endurance, strength, and body composition. Psychological outcomes included cancer-related fatigue, quality of life, self-efficacy, anxiety, and depression.

**Exclusion criteria:**

- Article type

Reviews and opinions

- Language

Other language

- Population

Healthy population, other chronic diseases, or cancer survivors

- Intervention

Acute virtual reality intervention (less than 1 week), no PA intervention

### Data Extraction

A data collection form was developed specifically for this review. It was used to capture the study reference with author, year of publication, study name, and location. We also extracted participant characteristics (sample size, age, and type of cancer), study design, methods used to assess the impact of exergaming, intervention program (frequency, intensity, temporality, time, and supervision), and outcomes (feasibility, adherence rate, and physiological and psychological effects).

### Study Quality Assessment

Study quality was assessed by one reviewer (RP) using a Cochrane tool and the Physiotherapy Evidence Database (PEDro) scale.

RoB 2 (version 2 of the Cochrane risk-of-bias tool for randomized trials) [31] was used for randomized controlled trials (RCTs). The risk of bias was assessed across 5 items: randomization process, deviation from the planned intervention, missing outcome data, outcome measurement, and selective reporting. These 5 domains were used to estimate an overall bias: “low risk,” “some concerns,” or “high risk.”

The PEDro scale is a valid scale for assessing risk of bias in clinical studies, regardless of design [32]. This tool provides a 10-point score through 11 “Yes-No” questions. The list of questions is available on the PEDro website. A lower score indicates poor-quality studies, and a higher score indicates high-quality studies.

## Results

### Study Selection

**Figure 1:**
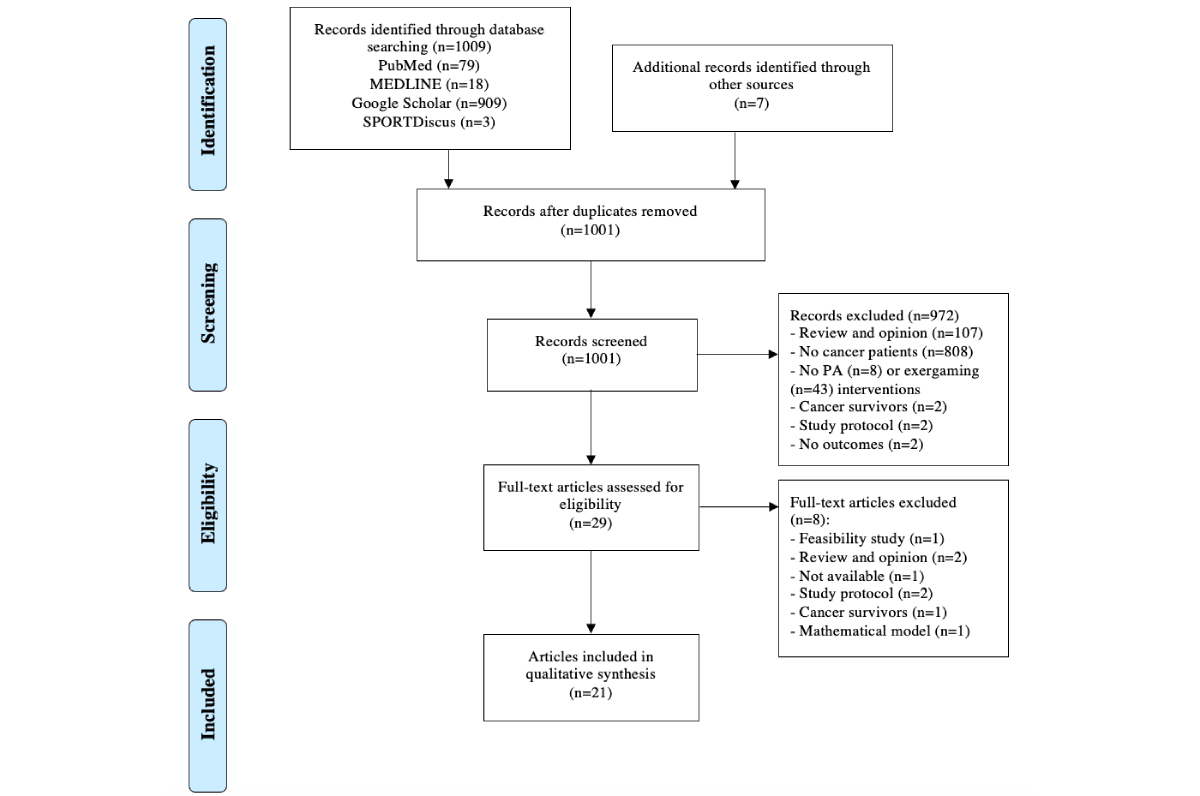
Flow diagram [29]. PA: physical activity.

On February 10, 2023, a total of 1009 articles were identified from PubMed (n=79), MEDLINE (n=18), Google Scholar (n=909), and SPORTDiscus (n=3). A total of 15 duplicates were removed, and 7 articles were manually added from reference checking of recent systematic reviews. Thus, 1001 articles were reviewed, and 972 were deleted after title and abstract screening. Reasons for exclusion were lack of the PA or exergaming intervention, lack of outcomes of interest, no patients with cancer, or patients who did not receive treatment. Review and opinion articles were also excluded. Therefore, after screening, 29 full-text articles were assessed for eligibility and 21 were retained and included in the qualitative synthesis. The 21 articles were combined into 17 trials. The study selection process is described in Figure 1, and the different steps are documented in Multimedia Appendix 1.

### Study Characteristics

No papers were published on the topic before 2013. Studies were published from 2013 to 2023 and conducted in several countries: Egypt, Saudi Arabia, Switzerland, Brazil (n=2), Finland, the United States (n=4), Turkey (n=2), Greece, Germany, Poland, Japan, and Denmark. Different study designs were adopted: 9 RCTs, 2 controlled quasi-experimental studies, 4 single-group studies, 1 case series, and 1 qualitative study.

In total, 379 patients with cancer participated in the studies (number of participants 3-70). The majority underwent treatment for hematologic, breast, lung, prostate, oral, or laryngeal cancer. The types and stages of cancer varied in all studies. Participants ranged in age from 3 to 93 years. Four studies included patients with pediatric cancer [33-36]. The duration of interventions ranged from 2 to 16 weeks, with a minimum of 2 sessions per week [37,38] and a maximum of 1 daily session [34,39]. Sessions were supervised in 10 studies [33,35-37,39-47], and 7 included home-based interventions [34,38,48-53]. Regarding exergames, 7 trials used Xbox Kinect, 8 trials used Nintendo Wii, and 2 trials created its own exergame and software. The characteristics of the studies and interventions are summarized in Table 1. Data extraction is available in Multimedia Appendix 2.

**Table 1:** Study characteristics.

Study design, country, reference	Cancer type	Population and age (years), n, mean (SD) or median (minimum-maximum)	System and exergames	Program duration (weeks)	Session frequency and duration	Intensity	Supervision, individual training (IT) or group training (GT)	Comparison group
Quasi-RCT^a^, Egypt [37]	Breast cancer	ExG^b^: 15, 54.07 (8.28); SPTG^c^: 15, 53.07 (7.24)	Nintendo Wii: tennis, triceps extension, and rhythmic boxing	4	2×/week for 30 minutes	NR^d^	Yes	SPTG: stretching and PNF^e^
RCT, Saudi Arabia [40]	Breast cancer	ExG: 30, 48.83 (7.0); SPTG: 28, 52.07 (7.48)	Xbox Kinect: Kinect Sports	8	5×/week	NR	Yes (GT)	SPTG: RES^f^
RCT, Switzerland [33]	Pediatric oncology	ExG: 22, 11.81 (2.41); MemG^g^: 23, 10.71 (2.48); CG: 24, 11.13 (2.47)	Xbox Kinect: Shape Up	8	3×/week for 45 minutes	RPE^h^: mean 4.35 (SD 2.23)/10	No	CG^i^: usual care; MemG: memory training
Controlled quasi-experimental, Brazil [41-43]	Various types (gastrointestinal tract, breast, abdominal and pelvic, or oropharyngeal)	ExG: 15, 57.13 (16.74); CAG^j^: 15, 63.29 (7.34); ExGh^k^: 15, 56.73 (11.94)	Xbox Kinect: Your Shape Fitness Evolved (Wall Breaker, Stomp It, and Run the World)	8-10	2-3×/week; ExG: mean 91.84 (SD 11.88) minutes/week; ExGh: mean 90.03 (SD 9.95) minutes/week	NR	Yes (IT)	N/A^l^
RCT, Turkey [38]	Breast cancer	ExG: 19, 50.84 (8.53); SPTG: 17, 51.00 (7.06)	Xbox Kinect: Dance Central and Kinect Sports	6	2×/week for 45 minutes	NR	No	SPTG: END^m^ and RES
Controlled quasi-experimental, Brazil [44,45]	Various types (gastrointestinal, breast, abdominopelvic, ovary, uterus, prostate, or oropharynx)	ExG: 10, 61.46 (8.79); ExGh: 10, 57.62 (7.57)	Xbox Kinect: Your Shape Fitness Evolved (Stomp It and Wall Breaker)	8-10	2-3×/week for 20-50 minutes	Light to moderate intensity	Yes (IT)	N/A
RCT, Finland [34]	Pediatric cancer	ExG: 17, 7.8 (3-16); CG: 19, 7.9 (3-15)	Nintendo Wii Fit: Hula Hoop or Jogging, Island Cycling, and Rhythm Kung-Fu	8	7×/week for 30 minutes	NR	No	CG: PA^n^ advice: 30 minutes/day
Single group, USA [48,49]	Early-stage non–small cell lung cancer	ExG: 7, 64.6 (6.5)	Nintendo Wii Fit Plus: Walking and balance games	16	Balance: 5×/week; Walking: 5×/week; W1^o^: 5 minutes/session incremented by 5 minutes/session each week if PSE^p^ >70%	Light intensity	No	N/A
Qualitative, Greece [35]	Pediatric cancer	ExG: 3, 5.66 (0.58)	Xbox Kinect: Kinect Sports 1 and 2, Kinect Adventures	12	3×/week for 30 minutes	NR	Yes (IT)	N/A
RCT, USA [50]	Prostate cancer	ExG: 8, 77.5 (6.7); SPTG: 6, 75.7 (9.5); CG: 5, 71.8 (5.0)	Nintendo Wii Fit	12	5×/week for 45 minutes	Light to moderate: HRr^q^: 60%-70%; RPE: 3-5/10	No	CG: usual care; SPTG: END and RES
Prospective randomized, Germany [46]	Hematologic cancer	ExG: 19, 56 (21-65); SPTG: 23, 56.5 (23-69)	Nintendo Wii Sport, Wii Fit, and Wii Balance Board	4	5×/week for 30-45 minutes	NR	Yes (IT)	SPTG: END and RES
Single group, Japan [47]	Hematologic cancer	ExG: 16, 66 (60-76)	Nintendo Wii Fit: Hula Hoop and Basics Step	Median: 23.5 days	5×/week for 20 minutes	METs^r^: Hula Hoop: 4; Basic steps: 3	Yes (IT)	N/A
RCT, Denmark [51]	Prostate cancer	ExG: 21, 67.6 (4.6); CG: 20, 69.8 (4.4)	Xbox Kinect: Your Shape Fitness Evolved, Sport and Adventure	12	3×/week for 60 minutes	NR	No	CG: usual care
Single group, United States [52]	Oral or laryngeal cancer	ExG: 8, 57.6 (13.3)	Nintendo Wii Fit: Wii Fit U	6	3-5×/week; W1-W3: 36 minutes/week; W4-W6: 40.1 minutes/week	HRpeak^s^: approx. 65%; RPE: 3-6/10	No	N/A
Single group, United States [53]	Advanced cancers	ExG: 4, 63.3 (8.7)	PAfitME (personalized exergame PA)	6	3-5×/week; W1-W3: 47.0 minutes/week; W4-W6: 81.2 minutes/week	RPE: 3-6/10	No	N/A
RCT, Poland [39]	Breast cancer	ExG: 9, 50.6 (12.6); CG: 7, 59.55 (7.85)	Virtual Therapeutic Garden	2	7×/week for 15 minutes	Light intensity	Yes (IT)	CG: usual care
Case series, Turkey [36]	Pediatric medulloblastoma	ExG: 5, 10.4 (3.5)	Nintendo Wii Fit Plus: Soccer heading, ski jumping, Penguin Slide, Ski Slalom, Balance Bubble	12	2×/week for 45 minutes	Light intensity	Yes (IT)	N/A

^a^RCT: randomized controlled trial.

^b^ExG: exergames group.

^c^SPTG: standard physiotherapy group.

^d^NR: not reported.

^e^PNF: proprioceptive neuromuscular facilitation.

^f^RES: resistance training.

^g^MemG: working memory training program group.

^h^RPE: Rating Perception of Exertion.

^i^CG: control group.

^j^CAG: remission patients.

^k^ExGH: exergame group with healthy volunteers.

^l^N/A: not applicable.

^m^END: endurance training.

^n^PA: physical activity.

^o^W1: week 1.

^p^PSE: personal self-efficacy.

^q^HRr: heart rate reserve.

^r^MET: metabolic equivalent task.

^s^HR peak: heart rate peak.

### Study Quality

Quality assessments of the randomized studies are presented in Table 2 and are available in Multimedia Appendix 3. Overall, the risk of bias ranged from low [40] to some concerns [33,38,39,46,50,51] to high [34]. This assessment depended primarily on knowledge of allocation, number of dropouts, lack of data, and heterogeneity of baseline results.

Quality ratings for nonrandomized studies are presented in Table 3. Scores ranged from 1 to 5. Non-RCTs, missing data, and dropouts limited quality.

**Table 2:** Risk of bias assessment for randomized trials.

Study	D1^a^	D2^b^	D3^c^	D4^d^	D5^e^	Overall
Basha et al [40]	Low	Some concerns	Low	Low	Low	Low
Benzing et al [33]	Low	Some concerns	Low	Low	Low	Some concerns
Feyzioğlu et al [38]	Low	Some concerns	Low	Low	Low	Some concerns
Hamari et al [34]	Low	High	Low	Low	Low	High
Sajid et al [50]	Some concerns	Some concerns	Low	Low	Low	Some concerns
Schumacher et al [46]	Low	Some concerns	Some concerns	Low	Low	Some concerns
Villumsen et al [51]	Low	Some concerns	Some concerns	Low	Low	Some concerns
Czech et al [39]	Low	Some concerns	Low	Low	Low	Some concerns

^a^D1: bias due to the randomization process.

^b^D2: bias due to deviations from intended interventions.

^c^D3: bias due to missing data.

^d^D4: bias in measurement of outcomes.

^e^D5: bias in selection of the reported results.

**Table 3:** Risk of bias assessment for nonrandomized trials.

Studies	Q1^a^	Q2	Q3	Q4	Q5	Q6	Q7	Q8	Q9	Q10	Q11	Score
Atef et al [37]	Y^b^	Y	N^c^	Y	N	N	N	Y	N	Y	Y	5
da Silva Alves et al [41], da Silva Alves [42] da Silva Alves [43]	Y	N	N	Y	N	N	N	N	N	Y	Y	3
de Oliveira et al [44] and de Oliveira et al [45]	Y	N	N	Y	N	N	Y	N	Y	Y	Y	5
Hoffman et al [48]	Y	N	N	N	N	N	N	N	Y	Y	Y	3
Hoffman et al [49]	Y	N	N	N	N	N	N	N	Y	Y	Y	3
Nani et al [35]	N	N	N	N	N	N	N	Y	Y	N	N	2
Tsuda et al [47]	Y	N	N	N	N	N	N	N	Y	N	Y	2
Wang et al [52]	Y	N	N	N	N	N	N	N	N	N	Y	1
Wang et al [53]	Y	N	N	N	N	N	N	N	N	N	Y	1
Tanriverdi et al [36]	Y	N	N	N	N	N	Y	Y	Y	Y	N	4

^a^Q1: Question 1.

^b^Y: yes.

^c^N: no.

### Feasibility and Adherence to Exergaming Interventions

Feasibility and adherence are presented in Table 4. On the whole, the exergaming interventions were feasible; 53.1% of patients agreed to participate. In addition, no adverse events related to AVG were reported. Regarding dropouts, 12 studies reported a rate of less than 20%, and 5 studies had 26.2% to 60% dropouts. The dropout rate was reduced by session supervision; supervised interventions had an 11.1% dropout rate [33,37-40,46,48,50,54] compared with 25.4% for those without supervision [34,35,41,42,44,47,51-53]. The dropout rate increased with age [41,43,44,46,47,50,52], male gender [50,51], and cancer aggressiveness [44,46,47,52,53]. Other reasons such as lack of time, travel difficulties, and patient death have also been reported [44,47,50,55].

Adherence rates were reported in only 6 studies. Three studies achieved an adherence rate of less than 70% [33,34,47], and 3 obtained a rate greater than 70% [36,48,49,52]. The number of studies is too small to provide convincing evidence of patient adherence to AVGs.

**Table 4:** Feasibility of intervention, dropouts, and adherence rate throughout intervention.

Study	Feasibility (participants/people meeting inclusion criteria)	Dropouts, n (%)	Adherence rate (total sessions completed [%])
Atef et al [37]	36/51	6 (16.7)	NR^a^
Basha et al [40]	60/112	2 (6.7)	NR
Benzing et al [33]	70/310	6 (8.6)	47.6% reached the desired 20 sessions
da Silva Alves et al [41], da Silva Alves [42], and da Silva Alves [43]	36/105	10 (18.2)	NR
Feyzioğlu et al [38]	40/67	4 (10.0)	NR
de Oliveira et al [44] and de Oliveira et al [45]	38/51	18/38 (47.4)	NR
Hamari et al [34]	36/47	1 (2.8)	50% the first week
Hoffman et al [48] and Hoffman et al [49]	7/10	0 (0)	First 6-week period: mean 96.6% (SD 3.4%, range 90%-100%); second 10-week period: mean 87.6% (SD 12.2%, range 59%-100%)
Nani et al [35]	NR	0 (0)	NR
Sajid et al [50]	19/31	Week 6 = 0 (0); week 12 = 6 (31.5)	NR
Schumacher et al [46]	42/49	11 (26.2)	NR
Tsuda et al [47]	NR	7/16 (43.8)	62%
Villumsen et al [51]	NR	5/46 (10.9)	NR
Wang et al [52]	10/85	2 (20)	First 3-week period: 75%; second 3-week period: 100%
Wang et al [53]	10/60	6 (60)	100%
Czech et al [39]	NR	0 (0)	NR
Tanriverdi et al [36]	NR	0 (0)	83.3%

^a^NR: not reported.

### Physiological Effects

Physiological outcomes are summarized in Table 5. The interventions based on AVGs showed varied physiological effects in patients with cancer.

PA levels were assessed in 6 studies using pedometers [48,50], accelerometers and diaries [34], or questionnaires [39,46,51]. Four studies found that AVGs did not significantly improve this parameter [34,46,50,51]. Hoffman et al’s study [48] indicated that AVGs could increase PA levels, but the authors did not present statistical analysis, and Czech et al’s study [39] indicated that AVGs increased PA levels significantly.

Muscular strength was assessed in 9 studies using hand dynamometers [38,40,46,47,50,52,53], electromyography [41-45], or a power bench [51]. After the intervention based on AVGs, strength was improved in 3 studies [38,40-43]. Five studies reported no significant effect of the AVG intervention [44,45,47,50-53], and Schumacher et al [46] demonstrated that patients had lost strength at the end of the intervention. In addition, 2 studies examined body composition [50,51]. The authors concluded that AVGs did not have a significant effect on body composition.

Aerobic capacity was assessed by a 2-minute walk test [46] or a 6-minute walk test [51-53]. Three of the 4 studies revealed a significant enhancement due to the AVG intervention [46,51-53].

In addition, physical function was assessed in 9 studies using questionnaires such as QuickDASH-9 (Quick Disabilities of the Arm, Shoulder and Hand) [37] and DASH [38,40] and tests such as the German Motor Test [33], Movement ABC-2 (Movement Assessment Battery for Children—Second Edition) [34], SPPB (Short Physical Performance Battery) [50], or Barthel Index [47]. Tanriverdi et al’s study [36] is based on the performances achieved in video games (ie, Fit Age in Nintendo Wii Fit Plus). Four studies showed a positive effect of AVGs on physical function [36-38,40], whereas the others did not report a significant effect.

**Table 5:** AVGs^a^ within-group effects on psychological and physiological outcomes.

References	Physiological outcomes						Psychological outcomes				
	PA^b^ level	Strength	Endurance	Physical function	BC^c^	QoL^d^		CRF^e^	Anxiety	Depression	SE^f^
Atef et al [37]	N/A^g^	N/A	N/A	+^h^ (*P*=.001)	N/A	N/A		N/A	N/A	N/A	N/A
Basha et al [40]	N/A	+ (*P*<.001)	N/A	+ (*P*<.001)	N/A	+ (*P*<.001)		N/A	N/A	N/A	N/A
Benzing et al [33]	N/A	N/A	N/A	=^i^ (*P*=.63)	N/A	N/A		N/A	N/A	N/A	N/A
da Silva Alves [41], da Silva Alves [42], and da Silva Alves [43]	N/A	+ (*P*<.01)	N/A	N/A	N/A	+ (*P*<.01)		+ (*P*<.01)	N/A	N/A	N/A
Feyzioğlu et al [38]	N/A	+ (*P*=.001)	N/A	+ (*P*=.001)	N/A	N/A		N/A	N/A	N/A	N/A
de Oliveira et al [44] de Oliveira et al [45]	N/A	+ Right deltoid (*P*=.01); = Left deltoid (*P*=.19)	N/A	N/A	N/A	N/A		+ (*P*=.001)	N/A	N/A	N/A
Hamari et al [34]	= (*P*<.05)	N/A	N/A	= (*P*<.05)	N/A	N/A		= (*P*<.99)	N/A	N/A	N/A
Hoffman et al [48] Hoffman et al [49]	+ (*P*=NR^j^)	N/A	N/A	N/A	N/A	N/A		+ (*P*=NR)	N/A	N/A	+ (*P*=NR)
Nani et al [35]	N/A	N/A	N/A	N/A	N/A	+ (*P*=NR)		N/A	N/A	N/A	N/A
Sajid et al [50]	= (*P*=.71)	= (*P*=.69)	N/A	= (*P*=.46)	= (*P*=.25)	N/A		N/A	N/A	N/A	N/A
Schumacher et al [46]	= (*P*=.09)	−^k^ (*P*=.02)	+ (*P*=.02)	N/A	N/A	+ (*P*=.001)		N/A	= (*P*>.05)	+ (*P*=.02)	N/A
Tsuda et al [47]	N/A	= (*P*=.28)	N/A	= (*P*=.58)	N/A	N/A		N/A	= (*P*=.05)	= (*P*=.22)	N/A
Villumsen et al [51]	= (*P*>.05)	= (*P*>.05)	+ (*P*=.02)	= (*P*>.05)	= (*P*>.05)	= (*P*=.61)		= (*P*=.15)	N/A	N/A	N/A
Wang et al [52]	N/A	= (*P*=.18)	= (*P*=.07)	N/A	N/A	N/A		+ (*P*=.03)	N/A	N/A	N/A
Wang et al [53]	N/A	= (*P*=NR)	+^l^	N/A	N/A	N/A		+^m^	N/A	N/A	N/A
Czech et al [39]	+ (*P*=.03)	N/A	N/A	N/A	N/A	N/A		N/A	N/A	+ (*P*=.02)	N/A
Tanriverdi et al [36]	N/A	N/A	N/A	+ (*P*=NR)	N/A	N/A		N/A	N/A	N/A	N/A

^a^AVG: active video game.

^b^PA: physical activity.

^c^BC: body composition.

^d^QoL: quality of life.

^e^CRF: cancer-related fatigue.

^f^SE: self-efficacy.

^g^N/A: not applicable.

^h^+: positive effect.

^i^=: no significant effect.

^j^NR: not reported.

^k^−: negative effect.

^l^Cohen *d*=0.6.

^m^Cohen *d*=0.7.

### Psychological Effects

Psychological outcomes are summarized in Table 5. Overall, AVG interventions maintained or improved psychological parameters.

Fatigue was assessed in 7 studies using the FACT-F (Functional Assessment of Cancer Therapy: Fatigue) scale, the Brief Fatigue Inventory scale, or the PedsQL (Pediatric Quality of Life Inventory) Multidimensional Fatigue subscale. Five AVG interventions led to an improvement in fatigue score [41-45,48,49,52,53], whereas Villumsen et al [51] and Hamari et al [34] reported no significant change.

Anxiety and depression were assessed in 2 studies using the HAD (Hospital Anxiety and Depression) scale. One study assessed depression through Beck Depression Scale. No significant results were found on anxiety [46,47]. However, Schumacher et al [46] and Czech et al [39] showed an improvement in the depression score.

Regarding QoL, 5 studies examined this outcome through interviews or questionnaires as well as FACT-BMT (Functional Assessment of Cancer Therapy: Bone Marrow Transplantation), FACT-P (Functional Assessment of Cancer Therapy: Prostate), or SF-36 (36-Item Short Form Health Survey). Four of them demonstrated that AVGs improved QoL in patients with cancer [35,40-42,46]. One study found no significant effect on this parameter [51].

Concerning the self-efficacy perception, Hoffman et al [48,49] used the Perceived Self-Efficacy for Fatigue Self-Management for Walking Duration questionnaire and a specific scale for balance activities. They demonstrated that the AVG intervention improved self-efficacy perception in patients with cancer.

### Comparison Between AVG and Standard Physiotherapy

Between-group comparisons are presented in Table 6. They revealed that AVGs induced greater benefits on QoL [46] than standard physiotherapy (SPT), as well as on vitality and general health, which are the subcomponents of QoL [40]. Similar results were reported regarding depression [46].

Concerning endurance, physical function, and strength, the data appeared controversial. Some studies mentioned an improvement in endurance [51] or physical fitness with AVGs [40], whereas others indicated the opposite [38] or no difference between these 2 approaches [37,38,46].

**Table 6:** Between-group comparisons on physiological and psychological outcomes.

References	Physiological outcomes						Psychological outcomes			
	PA^a^ level	Strength	Endurance	Physical function	BC^b^	QoL^c^		CRF^d^	Anxiety	Depression
Atef et al [37]	N/A^e^	N/A	N/A	ExG^f^=SPTG^g^ (*P*<.05)	N/A	N/A		N/A	N/A	N/A
Basha et al [40]	N/A	ExG<SPTG (*P*<.001)	N/A	ExG>SPTG (*P*<.001)	N/A	ExG=SPTG (*P*<.05); general health: ExG>SPTG (*P*<.001); vitality: ExG>SPTG (*P*=.006)		N/A	N/A	N/A
Benzing et al [33]	N/A	N/A	N/A	ExG=SPTG (*P*>.05)	N/A	N/A		N/A	N/A	N/A
Feyzioğlu et al [38]	N/A	ExG=SPTG (*P*=.30)	N/A	ExG<SPTG (*P*=.02)	N/A	N/A		N/A	N/A	N/A
Hamari et al [34]	ExG=SPTG (*P*=.38)	N/A	N/A	ExG=SPTG (*P*=.77)	N/A	N/A		ExG=SPTG (*P*<.05)	N/A	N/A
Sajid et al [50]	ExG<SPTG (*P*=NR^h^)	ExG<SPTG (*P*=NR)	ExG<SPTG (*P*=NR)	ExG<SPTG (*P*=NR)	ExG<SPTG (*P*=NR)	N/A		N/A	N/A	N/A
Schumacher et al [46]	ExG=SPTG (*P*<.05)	ExG=SPTG (*P*<.05)	ExG=SPTG (*P*<.05)	N/A	N/A	ExG>SPTG (*P*=NR)		N/A	ExG=SPTG (*P*<.05)	ExG>SPTG (*P*=NR)
Villumsen et al [51]	ExG=SPTG (*P*>.05)	ExG=SPTG (*P*=.22)	ExG>SPTG (*P*=.02)	ExG=SPTG (*P*=.08)	ExG=SPTG (*P*=.09)	ExG=SPTG (*P*=.61)		ExG=SPTG (*P*=.15)	N/A	N/A

^a^PA: physical activity.

^b^BC: body composition.

^c^QoL: quality of life.

^d^CRF: cancer-related fatigue.

^e^N/A: not applicable.

^f^ExG: exergames group.

^g^SPTG: standard physiotherapy group.

^h^NR: not reported.

## Discussion

### Principal Findings

AVGs are innovative tools in oncology. Safe, fun, and feasible PA interventions using AVGs have demonstrated beneficial effects on physical and psychological health.

In our systematic review, we reported that AVGs can help patients develop their endurance capacity because 3 of the 4 studies demonstrated an improvement of this outcome [46,51-53]. Increasing peak oxygen uptake values with AVGs could prevent the disease-associated loss of autonomy and allow the patient to live independently as a healthy individual. AVGs, through their repetitive and rapid movements, lead to PA of sufficient intensity to generate adaptations in pathological individuals, demonstrating the relevance of AVGs as a rehabilitation strategy [23,26,56].

AVGs presented mixed effects on patients’ physical functioning. When the practice of AVGs did not result in positive effects [34,47,50,51,55], the authors hypothesized that the intensity elicited by the AVGs would not be sufficient, except in the case of very deconditioned patients [46], or that the weekly duration of practice would be too short [46,55]. However, the second hypothesis seems less relevant, as 3 of the 4 studies reporting benefits offered only 2 sessions per week [36-38]. Another explanation could be the deterioration of patients’ health due to cancer treatments [44,47,48]. Among the studies reporting benefits [36-38,40], the protocols used differ in terms of frequency (2 [36-38] to 5 [40] sessions per week during 4 [37] to 12 weeks [36]), intensity (light to moderate [36-38,40]), and time (from 30 [37] to 45 minutes [36,38]), which prevents the definition of precise recommendations.

Contrary to SPT, AVGs do not significantly develop muscle mass and strength. In the 6 studies reporting no benefits, the AVGs proposed, whether commercial [46,47,50,51,53] or created [52], do not include muscle strengthening exercises. In the 3 studies reporting strength gain [38,42,44,45], patients used Xbox Kinect, suggesting that the type of movements performed during these AVGs may be advantageous in targeting this goal. Because muscle mass is predictive of patient life expectancy, it is essential to develop new AVGs with a muscle-strengthening component.

Among the psychological components, only CRF and QoL seem to be improved by the use of AVGs [35,40-42,44,46,48,52,53]. This was previously suggested by Ioannou et al [57] in their systematic review. Similarly, Ulas and Semin [2] also showed that virtual reality decreased perceived exercise intensity, reduced exercise stress, and improved perceived self-efficacy, thus helping patients to delay their fatigue threshold [2,58,59]. An improvement in sleep quality could also be achieved, leading to better recovery and less fatigue [2]. In our systematic review, sleep quality was not a primary outcome. Nevertheless, 2 studies evaluated the effects of exergames on this parameter using polysomnography and the Children’s Sleep Habit Questionnaire in children with acute lymphoblastic leukemia [60] and the Pittsburgh Sleep Quality Index in patients with breast cancer [39]. Both of these studies demonstrated the positive effects of the AVG intervention on sleep quality.

The physiological and psychological benefits in response to AVGs appear to be independent of increased PA levels. These results are surprising in view of the previous publications, showing that AVGs led to an increase in PA levels in various patients [23,27,56,61]. Several hypotheses can be proposed; wearing connected watches [34] is described as a behavior change technique [62] because it provides goal setting, action planning, and feedback [63] and could temporarily increase PA [58]. Hence, the first week’s measurement may be higher than usual because of the motivational dimension of the device. In contrast, at the end of the protocol, the PA level would be less modulated because of a gradual decrease in the motivation, possibly leading to monitor dropout [59,60,64]. This result can also be found with pedometers [48-50]. With respect to measures obtained using PA questionnaires, there may be a social desirability bias [65]. This bias may lead to overestimating the PA level on the initial assessment, but repetition of the measures would gradually reduce it [66]. An alternative explanation would be that participants decrease their home PA as a result of the increased PA achieved with the AVGs. This hypothesis is notably supported by Hoffman et al [48,49], who show that patients reduce their daily PA once they follow a walk program on the Wii Balance Board. Finally, in the study by Schumacher et al [46], patients with cancer complete the PA questionnaire before hematopoietic stem cell transplantation (T1), and then 7 days (T2), 14 days (T3), and 100 (T4) days after. The comparison is only made between T1 and T4, but we can assume that the level of PA drops after T1 in response to the treatments, explaining the lack of a significant difference between T1 and T4.

To sum up, in view of the physiological and psychological benefits observed, the use of AVGs in oncology appears to be relevant, particularly for patients who are far from PA practice sites and who can perform PA at home [13,14,67], and for those who are too weak or isolated because of the constraints of treatments (ie, sterile room). Our systematic review suggests that anticancer treatments [46,47,52-54] and advanced cancers [44,46,47,52,53] negatively influence patient adherence to interventions using AVGs. This result is also found for SPT [68]. Side effects (eg, fatigue, nausea, pain, or postoperative immobilization) may partially explain this finding. Moreover, AVG interventions appear to be better accepted by younger patients than by older patients. Familiarity and ease of use of technology may explain these results; older adults need tailored technology systems [69,70]. These results are reinforced by studies showing the influence of session supervision on patient adherence [33,36-40,48]. It would contribute to support patients in the use of new technologies and would therefore be more necessary than during SPT [67,71]. Finally, among the parameters of PA, intensity and frequency seem to be 2 key factors [38,41,42,49,51]. Based on the findings, the optimal recommendations would be to perform a minimum of 3 sessions of exercise per week at a light intensity.

### Study Limitations

Heterogeneity in settings, evaluations, and populations limits the ability to conclude on the effects of AVGs on specific cancer populations; therefore, only trends are presented in this review.

In addition, most of the nonrandomized trials presented low scores on the PEDro scale (from 1 to 5/10). Thus, some results should be viewed with caution because the study did not present statistical analysis [35,36,48,49] and the dropout rate was very high [44,45,47,53].

### Perspectives

Additional RCT and high-quality studies will be required to assess AVG feasibility with other patients with cancer and compare AVG intervention with SPT. In addition, further research will help define the optimal parameters of AVG interventions (ie, frequency, intensity, type, time, and supervision) based on patient characteristics and goals to be achieved. Also, future research should evaluate the effects of the AVG intervention combined with resistance training.

### Conclusions

The results of our review support the notion that AVGs can be recommended to patients undergoing cancer treatment, given the physiological and psychological benefits. The rates of engagement and adherence are similar to those found with SPT. However, as AVGs have no impact on body composition and muscle strength, we suggest combining AVGs with muscle strengthening exercises. Special attention should be paid to patients with advanced cancers and cancer cachexia to ensure that AVGs do not exacerbate weight and muscle loss.

## Appendix

Multimedia Appendix 1 Screening process.

Multimedia Appendix 2 Quality Assessment.

Multimedia Appendix 3 Data extraction.

## Abbreviations

AVG: active video game
CRF: cancer-related fatigue
FACT-BMT: Functional Assessment of Cancer Therapy: Bone Marrow Transplantation
FACT-F: Functional Assessment of Cancer Therapy: Fatigue
FACT-P: Functional Assessment of Cancer Therapy: Prostate
HAD: Hospital Anxiety and Depression
Movement ABC-2: Movement Assessment Battery for Children—Second Edition
PA: physical activity
PEDro: Physiotherapy Evidence Database scale
PRISMA: Preferred Reporting Items for Systematic Review and Meta-Analysis
QoL: quality of life
QuickDASH-9: Quick Disabilities of the Arm, Shoulder and Hand
RCT: randomized controlled trial
RoB 2: version 2 of the Cochrane risk-of-bias tool for randomized trials
SF-36: 36-Item Short Form Health Survey
SPPB: Short Physical Performance Battery
SPT: standard physiotherapy

## References

[1] Mina DS, Langelier D, Adams SC, Alibhai SMH, Chasen M, Campbell KL, Oh P, Jones JM, Chang E (2018). Exercise as part of routine cancer care. Lancet Oncol.

[2] Ulas K, Semin I (2021). The biological and motivational effects of aerobic exercise with virtual reality. Res Q Exerc Sport.

[3] Mishra SI, Scherer RW, Geigle PM, Berlanstein DR, Topaloglu O, Gotay CC, Snyder C (2012). Exercise interventions on health-related quality of life for cancer survivors. Cochrane Database Syst Rev.

[4] Campbell KL, Winters-Stone KM, Wiskemann J, May AM, Schwartz AL, Courneya KS, Zucker DS, Matthews CE, Ligibel JA, Gerber LH, Morris GS, Patel AV, Hue TF, Perna FM, Schmitz KH (2019). Exercise guidelines for cancer survivors: consensus statement from international multidisciplinary roundtable. Med Sci Sports Exerc.

[5] Cramp F, James A, Lambert J (2010). The effects of resistance training on quality of life in cancer: a systematic literature review and meta-analysis. Support Care Cancer.

[6] Dittus KL, Gramling RE, Ades PA (2017). Exercise interventions for individuals with advanced cancer: a systematic review. Prev Med.

[7] American Cancer Society (2022). Physical activity and the person with cancer internet. cancer.org.

[8] Friedenreich CM, Ryder-Burbidge C, McNeil J (2021). Physical activity, obesity and sedentary behavior in cancer etiology: epidemiologic evidence and biologic mechanisms. Mol Oncol.

[9] Booth FW, Laye MJ, Lees SJ, Rector RS, Thyfault JP (2008). Reduced physical activity and risk of chronic disease: the biology behind the consequences. Eur J Appl Physiol.

[10] Friedenreich CM, Stone CR, Cheung WY, Hayes SC (2020). Physical activity and mortality in cancer survivors: a systematic review and meta-analysis. JNCI Cancer Spectr.

[11] Brenner DR, Poirier AE, Grundy A, Khandwala F, McFadden A, Friedenreich CM (2017). Cancer incidence attributable to inadequate physical activity in Alberta in 2012. CMAJ Open.

[12] World Health Organization (2010). Global recommendations on physical activity for health. Internet.

[13] Avancini A, Tregnago D, Rigatti L, Sartori G, Yang L, Trestini I, Bonaiuto C, Milella M, Pilotto S, Lanza M (2020). Factors influencing physical activity in cancer patients during oncological treatments: a qualitative study. Integr Cancer Ther.

[14] Abo S, Denehy L, Ritchie D, Lin K, Edbrooke L, McDonald C, Granger CL (2021). People with hematological malignancies treated with bone marrow transplantation have improved function, quality of life, and fatigue following exercise intervention: a systematic review and meta-analysis. Phys Ther.

[15] Götte M, Kesting S, Winter C, Rosenbaum D, Boos J (2014). Experience of barriers and motivations for physical activities and exercise during treatment of pediatric patients with cancer. Pediatr Blood Cancer.

[16] Ross WL, Le A, Zheng DJ, Mitchell H, Rotatori J, Li F, Fahey JT, Ness KK, Kadan-Lottick NS (2018). Physical activity barriers, preferences, and beliefs in childhood cancer patients. Support Care Cancer.

[17] Solheim TS, Laird BJA, Balstad TR, Stene GB, Bye A, Johns N, Pettersen CH, Fallon M, Fayers P, Fearon K, Kaasa S (2017). A randomized phase II feasibility trial of a multimodal intervention for the management of cachexia in lung and pancreatic cancer. J Cachexia Sarcopenia Muscle.

[18] Arthur AE, Delk A, Demark-Wahnefried W, Christein JD, Contreras C, Posey JA, Vickers S, Oster R, Rogers LQ (2016). Pancreatic cancer survivors' preferences, barriers, and facilitators related to physical activity and diet interventions. J Cancer Surviv.

[19] Viana RB, de Oliveira VN, Dankel SJ, Loenneke JP, Abe T, da Silva WF, Morais NS, Vancini RL, Andrade MS, de Lira CAB (2021). The effects of exergames on muscle strength: a systematic review and meta-analysis. Scand J Med Sci Sports.

[20] Skip RA, Lange B, Suma EA, Bolas M (2011). Virtual reality and interactive digital game technology: new tools to address obesity and diabetes. J Diabetes Sci Technol.

[21] Evans E, Naugle KE, Kaleth AS, Arnold B, Naugle KM (2021). Physical activity intensity, perceived exertion, and enjoyment during head-mounted display virtual reality games. Games Health J.

[22] Peng W, Crouse JC, Lin J (2013). Using active video games for physical activity promotion: a systematic review of the current state of research. Health Educ Behav.

[23] Costa MTS, Vieira LP, Barbosa EDO, Mendes Oliveira L, Maillot P, Ottero Vaghetti CA, Giovani Carta M, Machado S, Gatica-Rojas V, Monteiro-Junior RS (2019). Virtual reality-based exercise with exergames as medicine in different contexts: a short review. Clin Pract Epidemiol Ment Health.

[24] Ainsworth BE, Haskell WL, Herrmann SD, Meckes N, Bassett DR, Tudor-Locke C, Greer JL, Vezina J, Whitt-Glover MC, Leon AS (2011). 2011 Compendium of physical activities: a second update of codes and MET values. Med Sci Sports Exerc.

[25] Pasco D, Bossard C, Buche C, Kermarrec G (2011). Utiliser les jeux vidéos actifs pour promouvoir l'activité physique. Sport Sci Rev.

[26] Lai B, Powell M, Clement AG, Davis D, Swanson-Kimani E, Hayes L (2021). Examining the feasibility of early mobilization with virtual reality gaming using head-mounted display and adaptive software with adolescents in the pediatric intensive care unit: case report. JMIR Rehabil Assist Technol.

[27] Qian J, McDonough DJ, Gao Z (2020). The effectiveness of virtual reality exercise on individual's physiological, psychological and rehabilitative outcomes: a systematic review. Int J Environ Res Public Health.

[28] Tough D, Robinson J, Gowling S, Raby P, Dixon J, Harrison SL (2018). The feasibility, acceptability and outcomes of exergaming among individuals with cancer: a systematic review. BMC Cancer.

[29] Moher D, Liberati A, Tetzlaff J, Altman DG (2009). Preferred reporting items for systematic reviews and meta-analyses: the PRISMA statement. PLoS Med.

[30] Pallot A (2019). Evidence Based Practice en rééducation. Démarche pour une pratique raisonnée.

[31] Sterne JAC, Savović J, Page MJ, Elbers RG, Blencowe NS, Boutron I, Cates CJ, Cheng H, Corbett MS, Eldridge SM, Emberson JR, Hernán MA, Hopewell S, Hróbjartsson A, Junqueira DR, Jüni P, Kirkham JJ, Lasserson T, Li T, McAleenan A, Reeves BC, Shepperd S, Shrier I, Stewart LA, Tilling K, White IR, Whiting PF, Higgins JPT (2019). RoB 2: a revised tool for assessing risk of bias in randomised trials. BMJ.

[32] de Morton NA (2009). The PEDro scale is a valid measure of the methodological quality of clinical trials: a demographic study. Aust J Physiother.

[33] Benzing V, Spitzhüttl J, Siegwart V, Schmid J, Grotzer M, Heinks T, Roebers CM, Steinlin M, Leibundgut K, Schmidt M, Everts R (2020). Effects of cognitive training and exergaming in pediatric cancer survivors—a randomized clinical trial. Med Sci Sports Exerc.

[34] Hamari L, Järvelä LS, Lähteenmäki PM, Arola M, Axelin A, Vahlberg T, Salanterä S (2019). The effect of an active video game intervention on physical activity, motor performance, and fatigue in children with cancer: a randomized controlled trial. BMC Res Notes.

[35] Nani S, Matsouka O, Theodorakis Y, Antoniou P (2019). Exergames and implications on quality of life in pediatric oncology patients: a preliminary qualitative study. J Phys Edu Sport.

[36] Tanriverdi M, Cakir FB, Mutluay FK (2023). Efficacy of a virtual reality-based intervention in children with medulloblastoma: case series. An Pediatr (Engl Ed).

[37] Atef D, Elkeblawy MM, El-Sebaie A, Abouelnaga WAI (2020). A quasi-randomized clinical trial: virtual reality versus proprioceptive neuromuscular facilitation for postmastectomy lymphedema. J Egypt Natl Canc Inst.

[38] Feyzioğlu Ö, Dinçer S, Akan A, Algun ZC (2020). Is Xbox 360 Kinect-based virtual reality training as effective as standard physiotherapy in patients undergoing breast cancer surgery?. Support Care Cancer.

[39] Czech O, Siewierska K, Krzywińska A, Skórniak J, Maciejczyk A, Matkowski R, Szczepańska-Gieracha J, Malicka I (2022). Virtual therapy complementary prehabilitation of women diagnosed with breast cancer-a pilot study. Int J Environ Res Public Health.

[40] Basha MA, Aboelnour NH, Alsharidah AS, Kamel FH (2022). Effect of exercise mode on physical function and quality of life in breast cancer-related lymphedema: a randomized trial. Support Care Cancer.

[41] da Silva Alves R, Iunes DH, Pereira IC, Borges JBC, Nogueira DA, Silva AM, Lobato DFM, Carvalho LC (2017). Influence of exergaming on the perception of cancer-related fatigue. Games Health J.

[42] da Silva Alves R, Iunes DH, de Carvalho JM, Menezes FDS, Silva AM, Borges JBC, Carvalho LC (2018). Effects of exergaming on quality of life in cancer patients. Games Health J.

[43] da Silva Alves R, Abdalla DR, Iunes DH, Mariano KOP, Borges JBC, Murta EFC, Michelin MA, Carvalho LC (2020). Influence of an exergaming training program on reducing the expression of IL-10 and TGF-β in cancer patients. Games Health J.

[44] de Oliveira PD, Iunes DH, Alves SA, de Carvalho JD, da Silva Menezes F, Carvalho LC (2018). Effects of exergaming in cancer related fatigue in the quality of life and electromyography of the middle deltoid of people with cancer in treatment: a controlled trial. Asian Pac J Cancer Prev.

[45] de Oliveira PF, Alves RDS, Iunes DH, de Carvalho JM, Borges JBC, Menezes FDS, Carvalho LC (2020). Effect of exergaming on muscle strength, pain, and functionality of shoulders in cancer patients. Games Health J.

[46] Schumacher H, Stüwe S, Kropp P, Diedrich D, Freitag S, Greger N, Junghanss C, Freund M, Hilgendorf I (2018). A prospective, randomized evaluation of the feasibility of exergaming on patients undergoing hematopoietic stem cell transplantation. Bone Marrow Transplant.

[47] Tsuda K, Sudo K, Goto G, Takai M, Itokawa T, Isshiki T, Takei N, Tanimoto T, Komatsu T (2016). A feasibility study of virtual reality exercise in elderly patients with hematologic malignancies receiving chemotherapy. Intern Med.

[48] Hoffman AJ, Brintnall RA, Brown JK, von Eye A, Jones LW, Alderink G, Ritz-Holland D, Enter M, Patzelt LH, Vanotteren GM (2013). Too sick not to exercise: using a 6-week, home-based exercise intervention for cancer-related fatigue self-management for postsurgical non-small cell lung cancer patients. Cancer Nurs.

[49] Hoffman AJ, Brintnall RA, Brown JK, von Eye A, Jones LW, Alderink G, Ritz-Holland D, Enter M, Patzelt LH, VanOtteren GM (2014). Virtual reality bringing a new reality to postthoracotomy lung cancer patients via a home-based exercise intervention targeting fatigue while undergoing adjuvant treatment. Cancer Nurs.

[50] Sajid S, Dale W, Mustian K, Kotwal A, Heckler C, Porto M, Fung C, Mohile SG (2016). Novel physical activity interventions for older patients with prostate cancer on hormone therapy: a pilot randomized study. J Geriatr Oncol.

[51] Villumsen BR, Jorgensen MG, Frystyk J, Hørdam B, Borre M (2019). Home-based 'exergaming' was safe and significantly improved 6-min walking distance in patients with prostate cancer: a single-blinded randomised controlled trial. BJU Int.

[52] Wang H-L, McMillan SC, Vijayakumar N, McDonald S, Huang L-T, Gwede C, Padhya T, Russell J, Vondruska K, Buck HG, Huang Y, Visovsky C (2019). A behavioral physical activity intervention to manage moderate and severe fatigue among head and neck cancer patients-pre-efficacy study in the national institutes of health ORBIT model. Cancer Nurs.

[53] Wang H-L, Donovan K, Padhya T, Szalacha L, Rechenberg K, Smith B (2021). Symptoms, biological stress response, and physical function after exergaming activities among advanced-stage cancer patients: 1461. Med Sci Sports Exerc.

[54] Tanriverdi M, Cakir E, Akkoyunlu M, Cakir F (2022). Effect of virtual reality-based exercise intervention on sleep quality in children with acute lymphoblastic leukemia and healthy siblings: a randomized controlled trial. Palliat Support Care.

[55] Benzing V, Eggenberger N, Spitzhüttl J, Siegwart V, Pastore-Wapp M, Kiefer C, Slavova N, Grotzer M, Heinks T, Schmidt M, Conzelmann A, Steinlin M, Everts R, Leibundgut K (2018). The Brainfit study: efficacy of cognitive training and exergaming in pediatric cancer survivors—a randomized controlled trial. BMC Cancer.

[56] Polechoński J, Nierwińska K, Kalita B, Wodarski P (2020). Can physical activity in immersive virtual reality be attractive and have sufficient intensity to meet health recommendations for obese children? A pilot study. Int J Environ Res Public Health.

[57] Ioannou A, Papastavrou E, Avraamides MN, Charalambous A (2020). Virtual reality and symptoms management of anxiety, depression, fatigue, and pain: a systematic review. SAGE Open Nurs.

[58] Brickwood K-J, Watson G, O'Brien J, Williams AD (2019). Consumer-based wearable activity trackers increase physical activity participation: systematic review and meta-analysis. JMIR Mhealth Uhealth.

[59] Esmonde K (2019). ‘There’s only so much data you can handle in your life’: accommodating and resisting self-surveillance in women’s running and fitness tracking practices. Qual Res Sport Exerc Health.

[60] Clawson J, Pater JA, Miller AD, Mynatt ED, Mamykina L (2015). No longer wearing: investigating the abandonment of personal health-tracking technologies on craigslist. http://dl.acm.org/citation.cfm?doid=2750858.2807554.

[61] Höchsmann C, Schüpbach M, Schmidt-Trucksäss A (2016). Effects of exergaming on physical activity in overweight individuals. Sports Med.

[62] Carey RN, Connell LE, Johnston M, Rothman AJ, de Bruin M, Kelly MP, Michie S (2019). Behavior change techniques and their mechanisms of action: a synthesis of links described in published intervention literature. Ann Behav Med.

[63] Düking P, Tafler M, Wallmann-Sperlich B, Sperlich B, Kleih S (2020). Behavior change techniques in wrist-worn wearables to promote physical activity: content analysis. JMIR Mhealth Uhealth.

[64] Quidu M (2019). L'auto-quantification de son activité sportive altère-t-elle la qualité de l'expérience vécue? Un scénario possible de l'abandon massif des pratiques de self-tracking.

[65] Butori R, Parguel B (2010). Les biais de réponse - Impact du mode de collecte des données et de l'attractivité de l'enquêteur.

[66] Coron C (2020). La boîte à outils de l'analyse de données en entreprise.

[67] Collado-Mateo D, Lavín-Pérez AM, Peñacoba C, Del Coso J, Leyton-Román M, Luque-Casado A, Gasque P, Fernández-Del-Olmo MÁ, Amado-Alonso D (2021). Key factors associated with adherence to physical exercise in patients with chronic diseases and older adults: an umbrella review. Int J Environ Res Public Health.

[68] Courneya KS, Friedenreich CM, Courneya KS, Friedenreich CM (2010). Physical activity and cancer: an introduction. Physical Activity and Cancer.

[69] Wang Hailiang, Zhang Jiaxin, Luximon Yan, Qin Mingfu, Geng Ping, Tao Da (2022). The determinants of user acceptance of mobile medical platforms: an investigation integrating the TPB, TAM, and patient-centered factors. Int J Environ Res Public Health.

[70] Seinsche Julia, de Bruin Eling D, Carpinella Ilaria, Ferrarin Maurizio, Moza Sotiria, Rizzo Francesco, Salatino Claudia, Giannouli Eleftheria (2023). Older adults' needs and requirements for a comprehensive exergame-based telerehabilitation system: a focus group study. Front Public Health.

[71] Forbes CC, Blanchard CM, Mummery WK, Courneya KS (2015). A comparison of physical activity preferences among breast, prostate, and colorectal cancer survivors in nova scotia, Canada. J Phys Act Health.

